# Exosomal Cargo in Ovarian Cancer Dissemination

**DOI:** 10.3390/cimb45120615

**Published:** 2023-12-07

**Authors:** Ekaterina Dzhugashvili, Svetlana Tamkovich

**Affiliations:** 1 V. Zelman Institute for Medicine and Psychology, Novosibirsk State University, 630090 Novosibirsk, Russia; 2 Institute of Chemical Biology and Fundamental Medicine, Siberian Branch of Russian Academy of Sciences, 630090 Novosibirsk, Russia

**Keywords:** exosomes, exosomal cargo, microRNA, proteins, liquid biopsy, ovarian cancer

## Abstract

Ovarian cancer (OC) has the highest mortality rate among all gynecologic cancers and is characterized by early peritoneal spread. The growth and development of OC are associated with the formation of ascitic fluid, creating a unique tumor microenvironment. Understanding the mechanisms of tumor progression is crucial in identifying new diagnostic biomarkers and developing novel therapeutic strategies. Exosomes, lipid bilayer vesicles measuring 30–150 nm in size, are known to establish a crucial link between malignant cells and their microenvironment. Additionally, the confirmed involvement of exosomes in carcinogenesis enables them to mediate the invasion, migration, metastasis, and angiogenesis of tumor cells. Functionally active non-coding RNAs (such as microRNAs, long non-coding RNAs, circRNAs), proteins, and lipid rafts transported within exosomes can activate numerous signaling pathways and modify gene expression. This review aims to expand our understanding of the role of exosomes and their contents in OC carcinogenesis processes such as epithelial-mesenchymal transition (EMT), angiogenesis, vasculogenic mimicry, tumor cell proliferation, and peritoneal spread. It also discusses the potential for utilizing exosomal cargo to develop novel “liquid biopsy” biomarkers for early OC diagnosis.

## 1. Introduction

Ovarian cancer (OC) ranks among the top ten causes of cancer mortality in women. According to Global Cancer Statistics, there were 313,959 new cases and 207,252 deaths diagnosed worldwide in 2020 [1]. The incidence of OC continues to rise annually, primarily due to the challenges of early diagnosis with only 20% of cases being detectable at stages I–II [2]. The challenge of early OC diagnosis stems not only from its asymptomatic development but also from the prolonged course of nonspecific symptoms, such as asthenic syndrome, weight loss, abdominal pain, urinary symptoms, etc. The primary methods for diagnosing OC and determining the possibility of optimal cytoreduction involve preoperative biopsy, histological analysis, and molecular study of tumor tissues. In cases where obtaining tissue samples is not feasible, clinical manifestations, ascites cytology, measurement of serum tumor marker levels (CA-125, CEA, HE4), and instrumental methods (CT, ultrasound, MRI) are used for diagnosis [3,4]. However, these biochemical markers are not reliable for the early detection of OC due to their limited specificity and sensitivity. Approximately 50–60% of OC cases do not exhibit elevated CA-125 levels [2], and approximately 30% do not show elevated HE4 levels [4,5].

Instrumental methods are commonly employed for OC diagnosis, but their effectiveness in detecting tumors smaller than 10 cm is limited. Transvaginal ultrasonography is the most common diagnostic, offering a sensitivity of 86% and a specificity of 91%. Additionally, it provides accurate visualization of the peritoneum, retroperitoneum, and inguinal lymph nodes, which is crucial for planning cytoreductive surgery [6].

High-field MRI is often used to determine the spread of malignant processes with a sensitivity of 91% and specificity of 88% [7]. One of the most promising instrumental diagnostic methods worth mentioning is positron emission tomography (PET). This method relies on the ability. One of the most promising instrumental diagnostic methods worth mentioning is positron emission tomography (PET). This method relies on the ability of actively dividing tumor cells to accumulate 18F-dGlu, which can be detected by PET [7]. Therefore, PET effectively addresses the issue of visualization of small-sized primary focus, metastases, and lymph nodes [3], and plays a crucial role in planning cytoreductive surgery [8,9]. Its sensitivity and specificity for detecting primary OC tumors are 67% and 79%, respectively. However, for recurrent OC, the sensitivity and specificity increased to 94.5% and 100%, respectively [7,9]. Despite the availability and widespread use of instrumental diagnostic methods, the detection of neoplasms remains a challenging endeavor.

One of the promising methods for early OC diagnosis is the detection of tumor biomarkers in extracellular vesicles (EVs) within biological fluids such as blood plasma and ascites, referred to as liquid biopsy. Liquid biopsy offers a comprehensive view of the molecular profile of the neoplasm since all tumor cells secrete EVs into the extracellular space.

Exosomes, which are lipid bilayer vesicles measuring 30–150 nm, have been shown to carry the tetraspanins CD9, CD63, and CD81 on their surface [10]. Exosomes are produced by all cells in the body, and the content of these vesicles varies depending on the condition of the donor cells. An elevated concentration of exosomes has been observed in tissues and biological fluids of various cancer patients, including those with OC [11,12]. The growing interest in exosome research and the search for new biochemical markers of malignant neoplasms are reflected in the increasing number of publications over the last decade, rising from 255 articles in 2013 to 2280 by the end of 2022. [https://pubmed.ncbi.nlm.nih.gov/, accessed on 1 April 2023].

## 2. Formation and Secretion of Exosomes

The process of exosome formation includes the following steps:

(1) Invagination of the plasmalemma and formation of early endosomes.
(2) Early endosomes mature into multivesicular bodies (MVBs), which contain intraluminal vesicles (ILVs) filled with various proteins, lipids, and nucleic acids. Notably, the cargo composition of ILVs is specific to the parent cell.
(3) Fusion of MVB with the plasmalemma results in the secretion of ILVs into the extracellular space. However, MVBs can also fuse with lysosomes or autophagosomes, leading to the degradation of ILVs [10,13].

Various published sources indicate that the regulation of exosome secretion involves proteins such as T-SNARE, SNAP23, and STX4 [13]. Additionally, the NSF and SNARE complexes play a role in the fusion of MVBs with the plasmalemma [14]. The Rab5 family GTPase is a key regulator of exosome formation and secretion. In particular, the conversion of Rab5 to Rab7, loss of Rab4, Rab11, Rab22, and the attachment of Rab9 are required during the maturation of MVBs [15]. Rab35 and Rab11, in turn, together with the GTPase activator protein TBC1D10A-C regulate the secretion of exosomes into the extracellular space [16]. Moreover, the influence of the Rab2b, Rab5a, and Rab9a proteins on exosome secretion has also been demonstrated [13]. In HeLa cell culture, Rab27a has been shown to play a role in the attachment of the MVB to the plasmalemma, while Rab27b is involved in the intracellular distribution of MVB [13]. The regulation of the p53 protein results in an increased expression of the *TSAP6* and *CHMP4C* genes, which, in turn, accelerates the maturation and secretion of exosomes. The activation of p53 and the overexpression of certain proteins are believed to be triggered by cellular DNA damage induced by oxidative stress which is necessary for signal transduction and the recruitment of other cells to mount a compensatory stress response. Since exosomes can fuse with the plasma membrane and transmit a signal to recipient cells, the increased formation and secretion of these vesicles under conditions of stress and hypoxia is not surprising [16].

The ESCRT complexes also play an important role in the formation of exosomes. Several types of these complexes have been identified: ESCRT-0, -I, -II, -III, and Vps4. All complexes closely interact with each other. ESCRT complexes are involved not only in the process of ILVs formation but also in the sorting of biologically active molecules in them. It has been observed that ubiquitinated proteins can be sorted into ILVs, a process that requires ESCRT-0. ESCRT-0, working in conjunction with clathrin-rich sites, captures ubiquitinated proteins and isolates them within the endosomal membrane. The ESCRT-I and -II complexes are involved in the invagination of the MVBs membrane and the formation of vesicles with clusters of ubiquitinated proteins inside. The ESCRT-III complex with its CHMP6 subunit, in turn, promotes the separation from the membrane and the release of vesicles within the MVBs by recruiting the CHMP4 protein. The ESCRT-0 complex requires the HRS protein, which is involved in recognizing monoubiquitinated proteins and sorting them into phosphatidylinositol-3-phosphate-rich endosomal compartments. HRS, together with Tsg101, recruits ESCRT-I [13,17]. Proteins involved in the ESCRT-dependent pathway of exosome formation could serve as promising targets for personalized therapy since inhibition of these proteins leads to decreased exosome secretion [10,13,18].

Another member of the ESCRT complex, the ALIX protein, plays an important role in the formation of exosomes. ALIX enhances the process of ILVs formation and promotes the integration of tetraspanins into exosomal membranes through interaction with HSPG1 [13].

An ESCRT-independent pathway of exosome formation has also been shown [18]. According to some studies, ceramides are an important part of the ESCRT-independent pathway due to their involvement in the invagination of the MVBs membrane and the formation of ILVs [15,19]. Ceramides are known to be formed by the action of nSMase from sphingomyelin. During this process, ceramide-rich platforms (CPRs) containing more than 20 ceramides can be formed. Some researchers propose that exosomes might transport CPRs or ‘mobile lipid rafts’ and activate specific signaling pathways in recipient cells that were previously active in donor cells. This mechanism could potentially play a pivotal role in the formation of pre-metastatic niches and carcinogenesis. Inhibition of nSMase has been found to lead to reduced exosome secretion both in vitro and in vivo [19]. Cholesterols also influence the process of MVB maturation: both fusion with the cell membrane, leading to the release of exosomes into the extracellular space, and fusion with lysosomes, leading to the degradation of MVBs, are possible. It has been demonstrated that exosomes containing a high concentration of cholesterol can fuse with the plasmalemma of the parent cell and are subsequently released into the extracellular space. Conversely, exosomes with a low cholesterol concentration tend to fuse with lysosomes and are subsequently destroyed [20].

Tetraspanins are also involved in exosome biogenesis within the ESCRT-independent pathway. Tetraspanins CD9, CD81, and CD63 are abundantly present in exosomes and are considered key markers of vesicles of exosomal origin [10,11]. CD63 is known to be involved in exosome biogenesis. A recent study showed a decrease in exosome secretion in HEK293 cell culture after CRISPR/Cas9 knockout of CD63. The interaction of CD9 and CD82 with ceramides leads to the enrichment of exosomes with β-catenin, which plays an important role in intercellular adhesion [13,21]. Furthermore, certain tetraspanins can reduce exosome secretion. For example, TSP6 triggers lysosomal degradation of MVB by interacting with exosomal SDC4 and syntenin. In addition, the loss of TSP6 in MCF-7 cell culture resulted in enhanced exosome secretion [22]. According to some data, both ESCRT-dependent and ESCRT-independent pathways can be simultaneously realized in cells [13].

**Figure 1:**
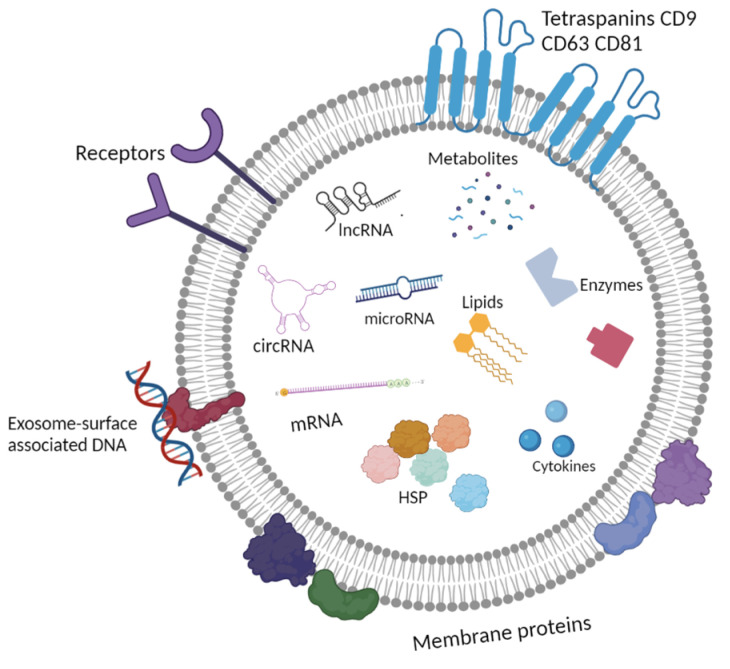
Scheme of the molecular content of the exosomes. Created with BioRender.com, accessed on 1 April 2023.

Exosomes have the ability to influence both nearby and distant cells as they spread through tissues via the blood and lymphatic system. As a result of exosomes spreading throughout the body, these vesicles can be found in saliva [23], blood plasma [24,25], ascites [26], urine [27], cerebrospinal fluid [28], breast milk [29], and tears [30]. It is known that 1 mL of blood from healthy donors contains 5–30 × 10^7^ exosomes [12,25,31]. Each exosome contains metabolites, lipids, proteins, and nucleic acids (Figure 1) [27,32].

Several studies have shown that exosomes can circulate in the blood by binding to the surface of blood cells [33,34,35]. Following this interaction, exosomes then bind to recipient cells through ligand-receptor interactions. Consequently, the membrane proteins of exosomes are important for delivering their cargo to target cells. For example, exosomes containing TSP8 and CD49d are more likely to bind to endothelial cells, while CD47-positive exosomes are more likely to bind to tumor cells [14]. Recipient cells internalize exosomes through various pathways, including clathrin-mediated endocytosis, lipid-transferase, caveolin-mediated endocytosis, phagocytosis, and micropinocytosis [13].

## 3. Morphology and Content of Exosomes

**Figure 2:**
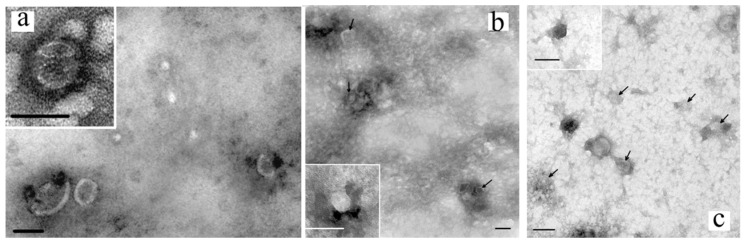
Total view of exosome preparation obtained from blood plasma of HFs (**a**), blood plasma of OCPs (**b**), ascitic fluid of OCPs (**c**). Inserts and arrows show exosomes. Scale bars correspond to 100 nm. Electron microscopy, negative staining using phosphotungstate acid [40].

The extensive use of transmission electron microscopy (TEM) has confirmed that exosomes are extracellular vesicles (EVs) characterized by low electron density and a cup-shaped morphology [36,37] in both the plasma and ascites of the OC patients (Figure 2). Additionally, cryo-electron microscopy has identified subpopulations of exosomes with single-, double-, and multi-layer membranes [36,38,39]. Although fundamental differences in the structure of exosomes in the blood of healthy women and cancer patients have been identified, data specific to OC are currently unavailable.

It is known that exosomes of different origins can differ in size. A recent study showed a significant difference between the exosomes of the IGROV1 cell culture (127 nm) and ES-2 cell culture (135 nm) [41]. In addition, the concentration of exosomes may differ depending on the biological fluid. The nanoparticle tracking analysis showed an increased concentration of exosomes in ascitic fluid compared to the blood plasma of the OC patients [26].

### 3.1. Lipidome of Exosomes

The lipid composition of exosomes is very similar to those in the plasma membrane of the parent cell. The curvature of exosomal membranes, which defines their size and biological functions, depends on their lipid content. The most commonly found lipids in exosomes are phosphoglycerolipids, sphingolipids, and sterols [10]. Exosomes exhibit a lipid composition that is notably enriched with phosphatidylserine, disaturated phosphatidylethanolamine, disaturated phosphatidylcholine, sphingomyelin, ganglioside GM3, and cholesterol when compared to their parent cells [42]. It’s important to note that the lipid subset of exosomes can vary among different subpopulations. For instance, the lipid composition of the CD61-positive exosomes significantly differs from that of other subtypes [43]. Mass spectrometry analysis of exosomes from 12 healthy donors and blood plasma revealed a higher lipid content in exosomes in comparison to plasma (244 and 191, respectively). Additionally, the concentration of triacylglycerol in exosomes was significantly higher than in plasma [44]. Furthermore, the exosomes’ lipid composition exhibits greater detergent resistance when compared to other extracellular vesicles [45].

The role of exosomal lipids in carcinogenesis is an area of active research. Lipidomic analysis of exosomes derived from the SKOV3 and HOSEPiC cell culture identified 1227 lipid species and 30 lipid classes. Notably, a significant difference in the lipid content of exosomes from the SKOV3 and HOSEPiC cell cultures was observed. The SKOV3-derived exosomes contained higher levels of GM3, zymosterol ester, lysophosphatidylinositol, lysophosphatidylserine, and cholesterol ester [46]. Exosomal lipids are also considered prospective biomarkers for tumorigenesis. For example, CerG3 was exclusively identified in the HOSEPiC-derived exosomes, while phosphatidylserine was specific to SKOV3-derived exosomes [46]. Such species specificity holds promise for the identification of new diagnostic biomarkers for liquid biopsy.

### 3.2. Proteome of Exosomes

The tetraspanins CD9, CD81, and CD63 are the most surface proteins on exosomes, making them markers for the exosomal nature of EVs. It is also known that the high heterogeneity of the exosomal proteins is due to the protein composition of parental cells as well as the conditions of their cultivation (hypoxia, acidic environment, etc.). According to the ExoCarta database (www.exocarta.org, accessed on 1 April 2023), which contains data from independent studies on the composition of exosomal proteins, lipids, microRNAs, and mRNAs, 9769 exosomal proteins have been identified as of 1 April 2023. The most common exosomal proteins and their functions are summarized in Table 1.

Proteomic analysis of exosomes obtained from the SKOV3 and HOSEPiC cell cultures revealed 659 universal proteins out of all 1433 identified exosomal proteins [46]. COX2 is one of the most abundant exosomal proteins whose increased expression is associated with hypoxia. Probably, the formation of tumor spheroids and metastasis process are caused by overexpression of this protein [47].

Additionally, a comparative proteomic analysis of blood plasma exosomes of 43 OC patients and 46 healthy donors showed the presence of 294 universal proteins for both groups. Furthermore, 69 unique proteins from blood plasma exosomes of the OC patients were identified [31].

Proteins implicated in carcinogenesis have been identified in the cargo of exosomes obtained from the blood plasma and ascitic fluid of OC patients. Specifically, proteins like ATF2, MTA1, ROCK1/2, and CD147 are involved in tumor angiogenesis, while GNA12, EPHA2, and COIA1 promote migration and metastasis. The Nanog protein plays a role in mediating the proliferation and invasion of tumor cells. Exosomes also contain Hsp90 and Hsc70, MHCI, and MHCII. Additionally, various enzymes have been detected in exosomes, such as phosphate isomerase, peroxiredoxins, aldehyde reductase, fatty acid synthase, and Dicer, which is involved in microRNA maturation, among others [48,49].

A recent study showed a significant increase in the level of exosomes of OC patients compared to healthy donors, as well as an increase in the level of LRP1 protein expression. LRP1 is known to be a low-density lipoprotein receptor family protein involved in the regulation of signaling pathway activation. It is assumed that LRP1 can control the expression of MMP2 and MMP9 through the activation of the ERK pathway and influence the migration of the OC cells in vitro and in vivo [50].

The important role of the PKR1 protein in the initiation of angiogenesis in vitro was demonstrated by the study of tumor-associated exosomes derived from A2780 and HO-8910 cell cultures. The PKR1 signaling pathway can be mediated by phosphorylation of STAT3. When the HUVECs cells were treated with the PKR1-positive exosomes, migration and tube formation were increased compared to controls [51].

It is known that ascitic fluid can be detected in small amounts even in the early stages of OC. As the tumor grows, ascites become a key component of the tumor microenvironment. Thus, proteomic analysis of exosomes derived from ascites may improve our understanding of OC dissemination as exosomes provide communication between cells and the tumor microenvironment. It has also been shown that up to 40% of the unique proteins of ascitic fluid are part of exosomes [52]. According to various published sources, ascitic fluid exosomes contain proteins such as CD24, EpCAM, CD171, MMP-2, MMP-9, uPA, MT1-MMP, ADAM10, ADAM7, ADAM17, CD151, TSPAN8 [26,53,54], as well as several other proteins and metabolites that mediate EMT, premetastatic niche formation, and peritoneal dissemination [55,56]. The overexpression of EpCAM, CD24, ADAM-10, ADAM17, CD82, CD151, and TSPAN8 in the exosomes of serum and plasma of OC patients compared to healthy women has also been shown to correlate with the stage and of the disease [26,54,57,58].

Given that these proteins are primarily found in tumor-derived exosomes and have a strong influence on the progression of OC, they can be considered promising targets for the treatment of OC as well as possible prognostic markers of the disease [26,54,57,58]. In addition, the proteins ZBED2, ZBTB20, ABCC3, and UHMK1, which are involved in cell cycle regulation and drug transport, were found in exosomes derived from ascitic fluid [59].

The formation of premetastatic niches is one of the key mechanisms of OC progression. Several studies have shown that exosomes secreted by the tumor play an important role in this process. For example, exosomes carry bioactive molecules such as TGFβ, TNFα, interleukins, MMPs, etc. that mediate EMT. Exosomes of cancer-associated fibroblasts are enriched with TGFβ and thus can activate the SMAD pathway in OC cells. This signaling pathway leads to enhanced cell migration and invasion. It’s well-documented that OC, like many other solid tumors, often occurs under hypoxic conditions. Hypoxia impacts various stages of carcinogenesis, including the formation and secretion of tumor-associated exosomes. For instance, hypoxia enhances exosome secretion by promoting the fusion of MVBs with the cell membrane [60]. Studies have demonstrated that OVCAR8, TR127, and TR182 cells grown under hypoxic conditions secrete a significantly higher number of exosomes compared to normoxic cells, primarily due to the upregulation of Rab27a and downregulation of Rab7, LAMP1/2, and NEU-1. Additionally, exosomes derived from cell lines of ovarian cancer patients’ ascites are characterized by an increased concentration of STAT3 and FAS in their cargo. These proteins have the potential to promote carcinogenesis and tumor dissemination. The cells derived from malignant ascites also exhibit an increased release of exosomes compared to immortalized cancer cells under normoxic conditions [61]. It should be noted that hypoxia can both enhance exosome secretion by the OC cells and alter the effects of exosomes on the tumor microenvironment. Exosomes also interact with tumor-associated macrophages and exosome-trained macrophages are known to promote OC cell migration and metastasis. Exosomes derived from hypoxic ovarian cancer epithelial cells play a role in mediating anti-inflammatory M2 polarization of macrophages, primarily through the suppressor of cytokine signaling (SOCS)4/5/STAT3 pathway. M2 polarization also influences the formation of pre-metastatic niches [48]. Additionally, exosomal proteins like ATF2, MTA1, and CD147 have been shown to induce tumor angiogenesis under hypoxic conditions [59].

The presence of soluble E-cadherin on the surface of exosomes mediates the binding of vesicles to VE-cadherin on endothelial cells, which leads to the activation of β-catenin and NF-κB pathways and the general induction of vasculogenesis [48].

Immunosuppression is crucial for tumor proliferation and metastasis. The ability of exosomes to influence this process in tumor cells has also been shown. For example, exosomes derived from ascitic fluid express the ganglioside GD3 on their surface. This molecule can interact with the T-cell receptor, resulting in the arrest of T cells. In addition to immunosuppressive mechanisms, the Fas ligand on the surface of the OC exosomes can suppress the T-cell receptor expression and mediate the T-cell apoptosis [48].

**Figure 3:**
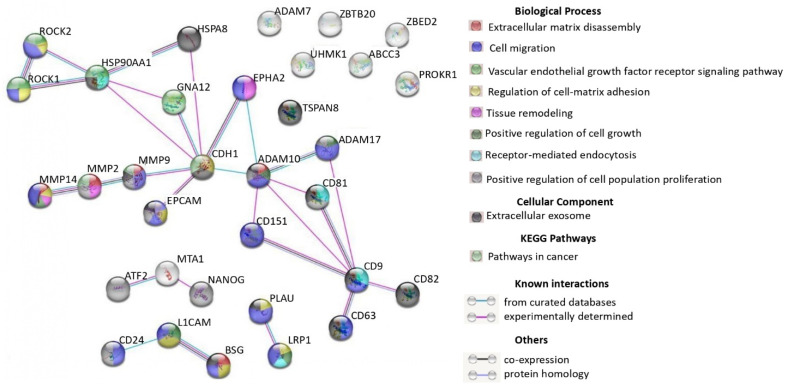
Interactions of proteins found in plasma exosomes and ascitic fluid of the OC patients that are involved in tumor proliferation and carcinogenesis.

The interactions of 33 exosomal proteins involved in tumor proliferation and carcinogenesis were analyzed using the STRING database (https://string-db.org/, accessed on 1 April 2023). The complex network organized by these proteins and the interactions between them are shown in Figure 3. Eighteen of the 33 proteins were found to interact with each other, with the vast majority of them involved in cell migration, regulation of cell-matrix adhesion, and tissue remodeling. In addition, ~20% of the proteins (ADAM7, ZBTB20, ZBED2, UHMK1, ABCC3, PROKR1, and TSPAN8) do not interact with any protein. Bioinformatic analysis revealed the interaction between ATF2, MTA1, and NANOG, which are involved in the processes of cell proliferation and angiogenesis [48,59].

### 3.3. Nucleic Acids Transported by Exosomes

In addition to proteins and lipids, exosomes carry functionally active nucleic acids. It has been shown that exosomes have DNA in their crown, but the proportion of such DNA does not exceed 0.025% of blood plasma DNA in healthy women [32]. It is also known that exosomes contain various types of RNA: mRNA, microRNA, long noncoding RNA, rRNA, tRNA, circRNA, etc. [62]. According to the Exocarta database (www.exocarta.org, accessed on 1 April 2023), exosomes are involved in the transport of more than 2838 microRNAs and 3408 mRNAs. Some researchers also consider lncRNAs and circRNAs as promising diagnostic biomarkers for liquid biopsy due to their ability to influence the carcinogenesis of tumors, including OCs.

#### 3.3.1. Long Non-Coding RNAs

Long noncoding RNAs (lncRNAs) are more than 200 bp in length [63] and are characterized by high variability and the ability to bind competitively to certain proteins or mRNAs. It has been shown that lncRNAs are involved in carcinogenesis, angiogenesis, metastasis, and drug resistance of tumors [64]. Comparative analysis of exosomes derived from the blood plasma of the OC patients, benign ovarian tumors, and healthy women revealed changes in the expression of 425 lncRNAs. Among these lncRNAs, the expression levels of FER1L6-AS2, LINC00470, LINC01811, CXXC4-AS1, LINC02343 and LINC02428 were significantly changed [65]. 

Evaluation of exosomes from the cisplatin-resistant A2780 cell culture showed that the level of lncRNA MEG3 typically downregulated in them. MEG3 acts as a carcinogenesis inhibitor and also decreases drug resistance. The lncRNA MEG3 sponges miR-214 is transported within exosomes and promotes cisplatin resistance of the OC cells [66].

LncRNAs ENST00000444164 and ENST0000043768 were identified in the SKOV3-derived exosomes. On HUVEC culture was shown, that these lncRNA play a key role in the restoration of endothelial cell migration, which was inhibited by TAM-derived exosomes. ENST00000444164 and ENST0000043768 are also involved in the phosphorylation of NF-κB. However, further research is required to determine the mechanism of NF-kB pathway regulation [67]. 

The elevated level of the exosomal MALAT1 in the blood serum of the OC patients correlates with aggressive disease and metastasis formation. The exosomes derived from both the OC cell cultures (SKOV3.ip1, HO8910.PM, SKOV3, and HO8910) and serum from patients with initial stage OC were enriched with MALAT1 to stimulate angiogenesis in HUVEC cell culture. In addition, the knockdown of MALAT1 in vitro and in vivo resulted in inhibition of angiogenesis. It is assumed that this effect is associated with the downregulation of some key genes related to angiogenesis [68].

The presence of lncRNA aHIF in blood serum exosomes of 62 OC patients was shown. The level of aHIF was significantly higher in OC patient-derived exosomes compared to HFs. Upregulation of this lncRNA is associated with an aggressive course of the disease and poorer overall survival. Thus, aHIF can be a potential biomarker of OC progression [69].

The lncRNA UCA1 was identified in serum exosomes obtained from 56 OC patients. Moreover, the level of UCA1 in serum exosomes obtained from the cisplatin-resistant group was significantly higher than that in the cisplatin-sensitive group. UCA1 was shown to contribute to tumor resistance to cisplatin through the UCA1/miR-143/FOSL2 axis. In addition, the UCA1 knockdown resulted in increased apoptosis of the cisplatin-resistant A2780-DDP and SKOV3-DDP cell lines after cisplatin treatment [70].

Examination of the plasma exosomes of the OC patients revealed increased levels of lncRNA SOX2-OT compared to healthy women. The exosomal SOX2-OT in TOV-21G and SKOV3 cell cultures promotes tumor cell migration, invasion, and proliferation, and decreased SOX2-OT levels resulted in suppression of these processes in vitro and in vivo. SOX2-OT is thought to affect tumorigenesis through regulation of the miR-181b-5p/SCD1 axis [71].

LncRNA ATB provides similar proangiogenic activity in HUVEC cell culture. ATB is thought to mediate carcinogenesis through the regulation of the miR-204-3p/TGFβR2 axis. In addition, it has been shown to stimulate tumor growth in vivo after treatment with exosomes derived from SKOV3 and A2780 [72].

**Figure 4:**
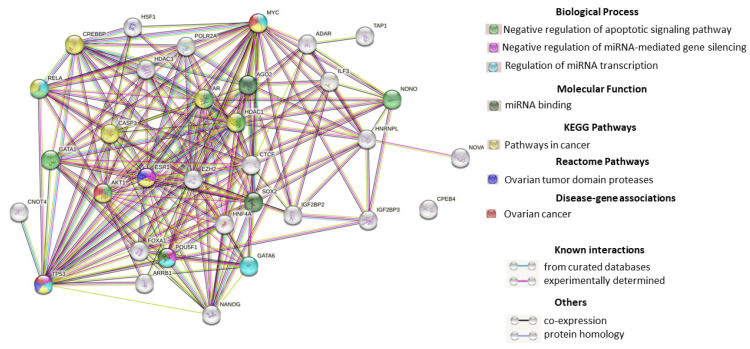
Interaction of proteins that are lncRNA targets within the exosomes of blood plasma and ascitic fluid of OC patients and involved in tumor proliferation and carcinogenesis.

The proteins with which lncRNAs interact were identified using the RNAInter v4.0 database. It was found that most of the target proteins are common to these lncRNAs. The interactions of 33 proteins were analyzed using the STRING database (string-db.org). The complex network organized by these proteins and the interactions between them are shown in Figure 4. 32 of the 33 proteins were found to interact with each other, with one-third of them involved in pathways in cancer or OC. Bioinformatic analysis revealed the interaction between AGO2 and SOX2, which are involved in the miRNA binding, MYC, RELA, GATA6, TP53, and POU5F1, which are involved in the regulation of miRNA transcription, and TP53, POU5F1, and ESR1 which are involved in the negative regulation of miRNA-mediated gene silencing.

#### 3.3.2. Circular RNAs

Circular RNAs (circRNA) are functionally active non-coding nucleic acids with a closed-loop structure [66], which makes them highly resistant to the action of RNases [56]. Because circRNAs have been discovered recently, our understanding of their involvement in tumor genesis is incomplete. It is assumed that circular RNAs can bind microRNAs [66] and proteins, which leads to the regulation of gene expression [59] and splicing processes [73].

Currently, the role of circRNAs in the processes of carcinogenesis and metastasis of OCs is of considerable interest. In particular, significant overexpression of Foxo3 circRNA has been shown in SKOV3-derived exosomes compared to HUM-CELL-0088-derived exosomes. Treatment of the SKOV3 cells with exosomes derived from cells overexpressing Foxo3 resulted in increased proliferation, migration, and invasion of SKOV3 cells. Foxo3 is thought to bind miR-422a and promote tumor progression through the miR-422a/PLP2 axis [74].

The Circ-0001068 circRNA expression has been shown to be significantly increased in exosomes derived from the serum of primary OC patients compared to those of healthy women. Circ-0001068 binds miR-28-5 and thereby promotes the PD1 expression on the surface of T cells. This leads to T-cell depletion and enhances OC growth and metastasis [75].

The level of circRNA051239 in exosomes derived from the plasma of the OC patients was also significantly elevated compared to healthy women. In a study using cultures of the high-metastatic SKOV3.ip and low-metastatic SKOV3 cells, exosomal circRNA051239 was shown to promote proliferation, invasion, and metastasis in low-metastatic SKOV3 cells. circRNA051239 is thought to bind miR-509-5p, which leads to overexpression of PRSS3 and promotes tumor progression [76].

The circNFIX circRNA is also capable of inducing carcinogenesis. For example, treatment of HUVEC cells with SKOV3-derived exosomes resulted in increased tube formation, enhanced proliferation, and migration of endotheliocytes. The circNFIX mediates the TRIM44 overexpression through the binding of miR-518a-3p. This leads to the regulation of the JAK/STAT1 pathway and stimulation of angiogenesis. Of note, a significant correlation was also found between the increased circNFIX and TRIM44 levels in tumor tissue samples from the OC patients and tumor size and FIGO stage [77].

The circRNAs may also inhibit carcinogenesis. In a study of exosomes derived from cancer-associated fibroblasts, the exosomal circIFNGR2 was shown to suppress proliferation, metastasis, and EMT in vitro and in vivo. CircIFNGR2 regulates the miR-378/ST5 axis, which leads to the inhibition of carcinogenesis [78].

#### 3.3.3. MicroRNAs

**Figure 5:**
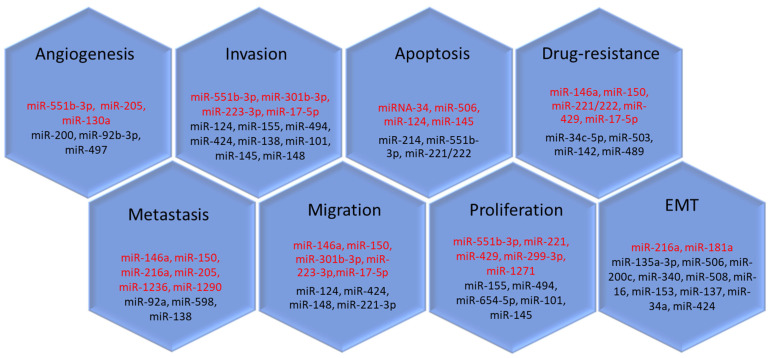
MicroRNAs involved in tumor dissemination of OC. The microRNAs that have a stimulating effect on carcinogenesis are marked in red, and the inhibitory effect is marked in black.

It is well known that exosomes carry microRNAs that affect various stages of carcinogenesis and metastasis. Many researchers have observed that the relative expression levels of the microRNAs change during the growth and progression of malignancies, including OC (Figure 5).

The targeted delivery of tumor-associated microRNA by exosomes to recipient cells enables not only additional stimulation of tumor growth but also reprogramming of tumor microenvironment and suppression of immune system cells (Figure 5, Table 2).

The concentration of exosomes in plasma and ascites is much higher than in tumor cells (~10^8^ versus 10) and is shown to be sufficient for the confident detection of several microRNAs in 1 mL of biological fluid [54,101]. Moreover, the revealed correlation of the exosomal microRNA levels in plasma and ascitic fluid inspires justified optimism that blood plasma is no less significant source of tumor markers than ascitic fluid [54].

## 4. Conclusions

Despite the effectiveness of instrumental methods in diagnosing OC, there are several limitations when it comes to detecting stage I disease, identifying cancer in situ, distinguishing between benign and malignant neoplasms, and conducting screening studies of both healthy women and cancer patients following courses of therapy. The high expectations associated with CA-125, CA 19-9, CEA, HE4, and αFP markers for OC detection have also given way to disappointment. To successfully detect neoplasms, there is now a recognized need to discover more sensitive and specific tumor markers for liquid biopsy and explore their combination with instrumental analysis methods. Furthermore, in the realm of modern personalized medicine, there is a pressing need for markers that can effectively predict aggressive tumor behavior and assess the efficacy of anti-cancer therapies. As tumor-secreted exosomes transport biopolymers critical for tumor growth and dissemination, analyzing the cargo of exosomes can provide insights into the molecular mechanisms that stimulate cancer cell proliferation, migration, invasion, angiogenesis, and immunosuppression. Research into the carcinogenic pathways involving proteins and non-coding RNAs (such as lncRNA, circRNA, and microRNA) present in exosomes circulating in the blood of OC patients holds the promise, in the near future, to not only enable the identification of effective markers for disease diagnosis and prognosis but also enhance the assessment of anti-tumor therapy. Furthermore, this research will serve as a foundation for the development of technology for obtaining vectors for gene therapy, built upon elements of natural transport systems.

## Figures and Tables

**Table 1:** Most frequently identified exosomal proteins due to ExoCarta data (as of April 2023).

No	Gene Symbol	Uniprot ID	Function
1	CD9	P21926	Membrane protein. Identified on membranes of oocytes and extracellular exosomes
2	HSPA8	P11142	Chaperone protein
3	PDCD6IP	Q8WUM4	Involved in sorting of cargo proteins of the MVBs for incorporation into ILVs
4	GAPDH	P04406	Modulates the organization and assembly of the cytoskeleton
5	ACTB	P60709	Protein that polymerizes to produce filaments
6	ANXA2	P07355	Calcium-regulated membrane-binding protein
7	CD63	P08962	Cell surface receptor for TIMP1 and plays a role in the activation of cellular signaling cascades AKT, FAK/PTK2 and MAPK
8	SDCBP	O00560	Involved in the trafficking of transmembrane proteins, exosome biogenesis, and tumorigenesis
9	ENO1	P06733	Involved in glycolysis, growth control, hypoxia tolerance, and allergic responses
10	HSP90AA1	P07900	Chaperone protein
11	TSG101	Q99816	The component of the ESCRT-I complex mediates the association between the ESCRT-0 and ESCRT-I complex
12	PKM	P14618	Catalyzes the final rate-limiting step of glycolysis generating ATP
13	LDHA	P00338	Interconverts simultaneously and stereospecifically pyruvate and lactate with concomitant interconversion of NADH and NAD+.
14	EEF1A1	P68104	Translation elongation factor that catalyzes the GTP-dependent binding of aminoacyl-tRNA (aa-tRNA) to the A-site of ribosomes
15	YWHAZ	P63104	Adapter protein implicated in the regulation of a large spectrum of signaling pathways
16	PGK1	P00558	Catalyzes one of the two ATP-producing reactions; acts as a polymerase alpha cofactor protein
17	EEF2	P13639	Catalyzes the GTP-dependent ribosomal translocation step during translation elongation
18	ALDOA	P04075	Plays a key role in glycolysis and gluconeogenesis; scaffolding protein
19	HSP90AB1	P08238	Chaperone protein
20	ANXA5	P08758	Acts as an indirect inhibitor of the thromboplastin-specific complex

**Table 2:** Exosomal microRNA in OC.

microRNA	Function/Biological Effect	Application	Expression	References
miR-205	Promotes angiogenesis via PTEN-AKT pathway and VEGFA regulation	Predict vascular mimicry and angiogenetic potential of tumor	Upregulated in exosomes of blood plasma of OC patients compared to HFs	[79,80,81]
miR-99	Promotes cell invasion and metastasis via fibronectin and vitronectin upregulation	Assessment of the aggressive course of the disease	Upregulated in exosomes of TYK-nu and HeyA8 OC cell lines compared to immortalized normal ovarian epithelial cell line.	[82]
miR-149	May enhance cisplatin resistance and EMT of OC via targeting CDKN1A and TIMP2.		Upregulated in exosomes of ascites of OC patients compared to peritoneal lavage of HFs.	[83,84]
miR-200a	May enhance tumor growth, metastasis, and chemosensitivity and also inhibit EMT via ZEB1/ZEB2 and TGF-β targeting		Upregulated in blood serum exosomes of OC patients compared to BOT patients.	[85,86,87]
miR-200b	Promotes invasion of OC cells via inhibiting M1 polarization of macrophages and KLF6 expression		Upregulated in exosomes of blood plasma of OC patients compared to HFs	[88,89]
miR-222-3p	Promotes OC progression via inducing M2-polarisation of macrophages by targeting SOCS3/STAT3 pathway		Upregulated in exosomes of blood serum of OC patients compared to HFs	[90]
miR-200c	Involved in cancer initiation and metastasis		Upregulated in blood serum exosomes of OC patients compared to BOT patients	[91]
miR-214	Binds to the 3′-UTR of PTEN and induces OC cell metastasis, proliferation, and cisplatin resistance via PTEN/Akt pathway regulation		Upregulated in exosomes of blood serum of platinum-resistant high-grade serous OC patients compared to BOT patients.	[92,93]
miR-221	Promotes proliferation and invasion of SKOV3 cells via BMF inhibition		Upregulated in exosomes of tumor-associated macrophages of OC patients	[94,95]
miR-1246 & miR-1290	Enhance the invasion and migration of OC cells via targeting RORα		Upregulated in malignant ascites-derived exosomes	[96]
miR-199a-5p	Inhibits carcinogenesis via HIF-2α, Wnt3a and β-catenin downregulation		Downregulated in exosomes from hypoxic UWB, HEY, A2780, and Anglne cell lines compared to normoxic exosomes from these cells	[97]
miR-940	Promotes proliferation and migration of OC via inducing M2-polarisation of macrophages		Upregulated in hypoxic SKOV3-derived exosomes compared to normoxic SKOV3-derived exosomes. Upregulated in ascites-derived exosomes compared to exosomes of peritoneal fluid of benign ovarian disease patients.	[98]
miR-429	Promotes tumorigenesis via inhibition of EPDR1 and activation of PI3K/AKT pathway. Low EPDR1 expression was correlated with tumor stage	Distinguish early and late stages of OC	Upregulated in blood serum exosomes of OC patients compared to HFs.	[99,100]
miR-34	Inhibit EMT via SNAIL, SLUG, and ZEB1 repression		Upregulated in blood serum exosomes of early-stage OC patients compared to late-stage OC patients	[101,102]
miR-6780	Promotes EMT and metastasis via regulating Notch and MAPK pathways	Predict overall survival and aggressiveness of OC	Upregulated in malignant ascites-derived exosomes	[103]
